# Memoranda on Poisons

**Published:** 1872-06

**Authors:** 


*Memoranda on Poisons.* By the late THOMAS HAWKES TANNER, M. D., F. L. S. Third and completely revised edition. Philadelphia: Lindsey & Blakiston 1872.

These two little books will be perhaps, more readily received by the student, who is preparing for examination, or who wishes to refresh his mind on some of the salient points without reading over the whole subject, than by the practitioner, who has or should have the whole subject stored up in his mind.

For any one however who is forgetful for the time being of some technical term, or especial position, or presentation in obstetrics Dr. Rigby's memoranda is without doubt just the thing to set him right.

Often in the hurry and excitement of the moment the young practitioner who is called to a case of poisoning is forgetful of the proper mode of treatment; to such no book of its size that we are acquainted with will be of more aid than the memoranda of poisons by Dr. Tanner. Although we should look with disfavor upon any physician who should be in the habit of relying upon any pocket aid to his memory, yet we can imagine of instances where in these little books would be of considerable aid.

Both books have been revised and to a large extent re-arranged and are presented to the profession in a very engaging form.

